# Suicide

**Published:** 1871-02

**Authors:** 


### SUICIDE.

If a man in an instant drives a dagger to his heart, he is branded as a suicide. If he arranges to drive it in the fraction of an inch daily, until it is up to its handle, it is not the less a suicide. But suppose the unfortunate individual should say that he did not know it would kill him, he would be a suicide still, or a fool, or both.

If a man lays himself across the rail for the locomotive, and he is cut in two, it would not answer for him to have said that he did not know the engine was coming along, and he must come under the ban of the suicide. If a man drinks too much every day, or eats too much every day, until he finally dies a drunkard or a glutton, small pity will be felt for him, if he should say with his last breath he did not know that he was killing himself.

That man is really a suicide who loses health and life by the want of a wise, judicious care of himself. One of the greatest theological minds of the age was lamented a few years ago, among Presbyterians, as too early lost. A biographer wrote of him that he had a kind of contempt for physicians. He had been ailing some time; rode out one day, thinking he was getting well; the day following he died; not less a suicide than another clergyman, quite deaf, who, a few days ago, was walking on a railroad track, when the locomotive walked over him. Few more useful men have been lost to the church of late years; his success as a minister was steadily increasing until the last year of his life gave him sixty or seventy additions to his church, by "examination." It was said of him, "He was a man of high culture and endowments. His mind was logical. He was profound and exhaustive in argument, and had few equals in rhetorical expression," and yet had not logic enough to keep out of the way of a locomotive. A cultivated man and profound, of great endowments, and deaf as a post, walking on a railroad track! Whether he had no common sense, or had a purpose, the General Judgment only can decide. He either did not make use of his talent of culture and endowment, and now stands before his Maker in default, or he did make use of them in cool calculation, and stands there as an A No. 1 criminal. In either case his life was uselessly sacrificed, to the loss of society, of the church, and of the world. He was either criminally negligent or deliberately criminal.

The time has passed, reader, for canonizing every man who dies. The only true biographer is God. It is in the grand old Bible alone that the histories of men are given with exact justice. It admits of debate whether it is not better to praise a man while he still lives, to encourage him to do better, and abuse him after he is gone, when it can't hurt his feelings. This will teach the living the wholesome lesson that their short-comings are certain not to be covered up when they are dead.

It is said in the same panegyric of the deaf man, "For many years he had been deprived of his hearing, and it was on this account he lost his life;" which means that the loss of his hearing was from an agency outside of himself, and that consequently he was not responsible for his premature death, when really it is next to a certainty that his deafness arose from a logic alike to that which led him to walk on a railroad track. But the unpardonable stupidity of men in walking on rail tracks is only equaled by the persistence of the people in trying to replenish kerosene lamps with oil while they are burning.

